# Sperm of Fruit Fly *Drosophila melanogaster* under Space Flight

**DOI:** 10.3390/ijms23147498

**Published:** 2022-07-06

**Authors:** Irina V. Ogneva, Yulia S. Zhdankina, Oleg V. Kotov

**Affiliations:** Cell Biophysics Laboratory, State Scientific Center of the Russian Federation Institute of Biomedical Problems of the Russian Academy of Sciences, 76a, Khoroshevskoyoe Shosse, 123007 Moscow, Russia; juliaszd@yandex.ru (Y.S.Z.); kotov2710@gmail.com (O.V.K.)

**Keywords:** sperm motility, space flight, dynein, cytoskeleton, cellular respiration

## Abstract

Studies of reproductive function under long-term space flight conditions are of interest in planning the exploration of deep space. Motility, including the use of various inhibitors, cellular respiration, and the content of cytoskeletal proteins were studied, assessing the level of expression of the corresponding genes in spermatozoa of *Drosophila melanogaster*, which were in space flight conditions for 12 days. The experiment was carried out twice on board the Russian Segment of the International Space Station. Sperm motility speed after space flight, and subsequently 16 h after landing, is reduced relative to the control by 20% (*p* < 0.05). In comparison with the simulation experiment, we showed that this occurs as a result of the action of overloads and readaptation to the Earth’s gravity. At the same time, cellular respiration, the content of proteins of the respiratory chain, and the expression of their genes do not change. We used kinase inhibitor 6-(dimethylamino)purine (6-DMAP) and phosphatase inhibitors; 6-DMAP restored the reduced the speed of spermatozoa in the flight group to that of the control. These results can be useful in developing a strategy for protecting reproductive health during the development of other bodies in the solar system.

## 1. Introduction

In the future, humanity will most likely face the need for long space flights and the development of other bodies, both in the solar system and beyond. In this case, the maintenance of the species will occur in conditions of weightlessness and/or with a changed gravity. Therefore, in order to develop effective methods for protecting reproductive potential, studies of gametogenesis and embryogenesis under space flight conditions are required.

One of the most convenient objects for studying the effect of gravity on gametogenesis and the development of a multicellular organism over several successive generations is the fruit fly *Drosophila melanogaster*. Therefore, a number of experiments under space flight conditions were carried out on different lines of *Drosophila melanogaster* [1,2]. For example, after a 7-day Challenger flight (1985, USA), the number and size of eggs of adult *Drosophila melanogaster* of the Oregon R line increased, and approximately 25% of the embryos did not develop into adults [3], although the same authors were unable to reproduce these effects in improving fly oxygenation [4]. After a 44.5-day flight of the Foton-M No. 4 satellite and a subsequent 12-day flight aboard the Russian Segment of the International Space Station (2014, Russia), we also received third-fifth generation *Drosophila melanogaster* adults without embryonic development anomalies, although we did not estimate the percentage of adult appearance individuals from laid eggs [5]. However, in the same flies after landing, after three generations of exposure to the Earth’s gravity in the ovaries, the content of cytoskeletal proteins and the corresponding mRNAs remained significantly reduced [6]. In spermatozoa, the frequency of dominant lethal mutations under space flight conditions was higher than in the control, and especially in immature forms, spermatids and spermatocytes [7], which can reduce the number of mature spermatozoa. The foregoing suggests that the early stages of embryogenesis and the state of gametes may be especially dependent on the external mechanical field and space flight conditions.

Indeed, when changing gravity, it was shown that cells that were cultured under these conditions had changes in cellular profile, probably due to changes in the structure of the cytoskeleton [8,9,10]. The latter may be associated with changes in the level of expression of genes encoding cytoskeletal proteins and corresponding participants in signaling cascades [11,12,13]. Reorganization of the cytoskeleton can also lead to a change in the differentiation potential of stem cells, which was observed when modeling the effects of weightlessness [14,15]. Therefore, it can be expected that in eggs and sperm cells, as well as in other cell types, changes in the structure of the cytoskeleton and gene expression can appear under conditions of altered gravity.

The spermatozoa of various animal species differ considerably from each other. However, when comparing the male sperm cells of standard subjects of experimental biology, mice and flies, it should be noted that their main structural and functional parameters are quite similar (for example, the structure of the spermatozoon, the structure of the axoneme, and high homology structural proteins of the axoneme).

It has been shown that space flight conditions, as well as simulated weightlessness, reduce the number of mature mice [16,17,18]. Moreover, a decrease in the speed of movement and the proportion of motile spermatozoa, as well as change in the content of cytoskeletal proteins and the mRNA of the corresponding genes, are noted [19,20,21].

For flies under simulated weightlessness, it was shown that, unlike mice, the speed of spermatozoa increases in short experiments [22]. Moreover, the difference in the reactions of spermatozoa of flies and mice to simulated microgravity is apparently due to differences in regulation of the phosphorylation/dephosphorylation of motor proteins [23].

However, space flight has a number of factors, the combined effect of which may differ from the effect of weightlessness alone. When flying in low orbits, as in the International Space Station (ISS), the contribution of ionizing radiation is not as significant. The main effect in this case is exerted by weightlessness and overloads during launch and landing.

Therefore, the aim of this study was to assess the state of the spermatozoa of the fruit fly *Drosophila melanogaster* after the full cycle of spermatogenesis which occurred under the conditions of a real space flight aboard the Russian Segment of the International Space Station (RS ISS). We removed the testis and froze them at the landing site and 16 h later in the laboratory, where, in addition to freezing, we had the opportunity to conduct in vivo studies assessing sperm motility and cellular respiration. Accordingly, to determine the contribution of each factor (launch overloads, weightlessness, landing overloads, and readaptation period to 1 g for 16 h) to the results obtained, simulation studies on Earth were carried out synchronous to the space flight.

## 2. Results

### 2.1. Experimental Design

Eleven days before the start of a real or simulated space flight, fifty pairs of virgin imagoes of the fruit fly *Drosophila melanogaster* of the Canton S line, 2 days old, were placed in 50 mL Falcon-type test tubes with an air-permeable lid containing a standard medium for breeding *Drosophila* with the following composition: water—1000 mL, agar-agar—7 g, granulated sugar—40 g, semolina—40 g, baker’s yeast—25 g, and propionic acid—10 mL. The flies laid eggs for 1 day and were then removed from the test tubes, leaving only eggs. Next, these test tubes were exposed to either a real space flight or a simulation experiment on Earth. The flies took off on the second day of their exposure to conditions of real or simulated weightlessness.

The “Cytomehanarium” space experiment (as part of a real space flight aboard the RS ISS) and a simulation experiment were carried out twice with four biological replicas each (at least three biological replicas were obtained for each determined parameter). Space flights were carried out from 5 October 2021–17 October 2021 (ISS-65 expedition) and 18 March 2022–30 March 2022 (ISS-66 expedition). Samples were placed into the spaceship during the latest access for payload, approximately 8 h before launch. All temperature and atmosphere parameters were controlled before launch and during the flight to the ISS.

For the simulation experiment, a controller (Gravite^®^, GC-US-RCE010001, Space Bio-Laboratories Co., Ltd., Hiroshima, Japan) was used, which facilitates reproducing G-forces during launch and landing, as well as weightlessness based on the principle of operation of a random positioning machine [24].

Overloads at the launch were simulated according to the cyclogram of the real launch of the Soyuz spacecraft: 2 g—50 s, 2.5 g—18 s, 3 g—50 s, 2 g—168 s, 2.5 g—120 s, 3 g—120 s, and then—0 g within 12 days. Landing overloads were also reproduced according to the landing sequence of the Soyuz descent vehicle: 3 g—425 s, 1 g—200 s, 3 g—5 s, 1 g—625 s, kick, and then on Earth.

In a real space flight and the corresponding synchronous control, the testes of some males were removed at the landing site and immediately frozen for subsequent determination of the content of proteins and mRNA. In the remaining males, the testes were removed in the laboratory (16 h after landing); some were frozen for subsequent isolation of protein and mRNA, and some were used in vivo experiments to determine the parameters of sperm motility and cellular respiration.

As it was impossible to carry out in vivo studies at the landing site in the steppe, the same procedures were carried out in the simulation experiment; in addition, in vivo studies were carried out at the quasi-“landing site” to identify the role of overloads and the early period of readaptation to the studied parameters.

**Figure 1:**
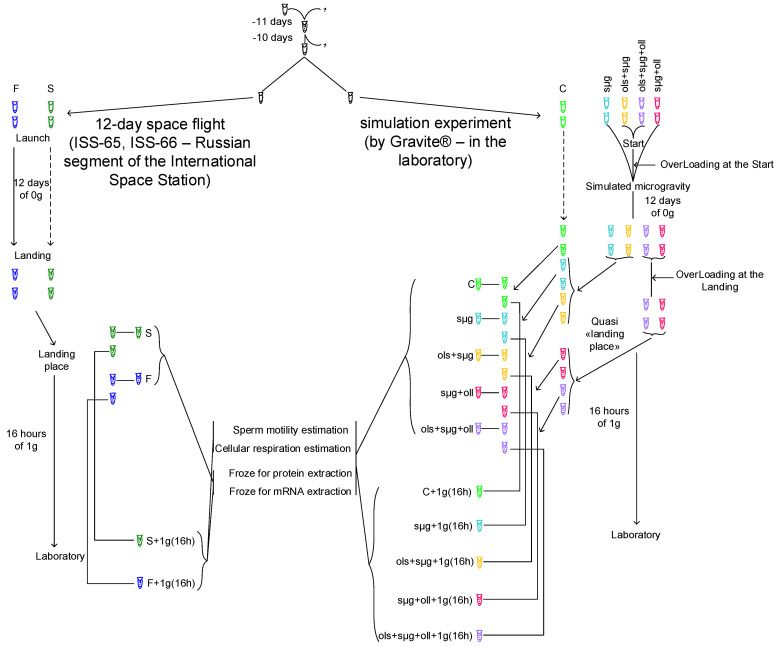
**Experimental design: the timeline of the experiment and points of the measurements.** Timeline of the flight: overloading at the start 2 g—50 s, 2.5 g—18 s, 3 g—50 s, 2 g—168 s, 2.5 g—120 s, 3 g—120 s; weightlessness 0 g—12 days; overloading at the landing 3 g—425 s, 1 g—200 s, 3 g—5 s, 1 g—625 s, kick; and readaptation 1 g—16 h. A portion of the samples from flight group F and synchronous control S were frozen at the landing site; additional portions were tested for sperm motility and cellular respiration in the laboratory and then also frozen. For the simulation experiment all tests were made at the quasi “landing site” and after 16 h of 1 g conditions.

Fourteen study groups were formed (Figure 1); seven groups were examined immediately after the end of the flight (real or simulated) and seven groups were examined after 16 h in the Earth’s gravity. The first seven groups were:

C—Control—the control group, which was kept under standard laboratory conditions;

sµg—Simulated MicroGravity—a group that was in simulated microgravity for 12 days;

ols+sµg—OverLoading at the Start + Simulated MicroGravity—a group subjected to starting overloads and subsequent 12-day simulated microgravity;

sµg+oll—Simulated MicroGravity + OverLoading at the Landing—a group that was in simulated microgravity for 12 days and then subjected to overloads during landing;

ols+sµg+oll—OverLoading at the Start + Simulated MicroGravity + OverLoading at the Landing—a group that reproduces the entire space flight cyclogram, including overloads at the launch, simulated weightlessness for 12 days, and overloads at landing;

S—Synchronous control—the control group (in fact, a “duplicate” of the space flight), which was contained in the same packing as the space flight group, and accompanied the space flight group until loading into the spacecraft at launch and immediately after the spacecraft landed at the landing site;

F—Flight—a group that experienced real space flight aboard the Russian Segment of the ISS (launch overloads, real weightlessness for 12 days, landing overloads).

The same seven group classifications, with the addition of a 16 h readaptation to the Earth’s gravity 1g(16h) are: C+1g(16h), sµg+1g(16h), ols+sµg+1g(16h), sµg+oll+1g(16h), ols+sµg+oll+1g(16h), S+1g(16h), and F+1g(16h).

All groups had the same temperature regime, which was observed according to the temperature chart of the space flight group F.

All experimental procedures were approved by the Commission on Biomedical Ethics of the Institute of Biomedical Problems (IBMP), the State Scientific Center of the Russian Federation, and the Federal State Budgetary Institution of Science (Minutes No. 521 dated 25 September 2019).

### 2.2. Sperm Motility

Studies of sperm motility at the landing site of the Soyuz descent vehicle with cosmonauts on board (147 km southeast of Zhezkazgan, Kazakhstan) were not possible due to organizational difficulties.

The speed of movement of spermatozoa between the control groups, C, C+1g(16h) and S+1g(16h) within each series (without the addition of inhibitors, with the addition of sodium orthovanadate, calyculin A, and 6-DMAP) did not differ.

The addition of Tyr phosphatase inhibitor 200 µM sodium orthovanadate completely stopped sperm motility in all study groups, including controls.

**Figure 2:**
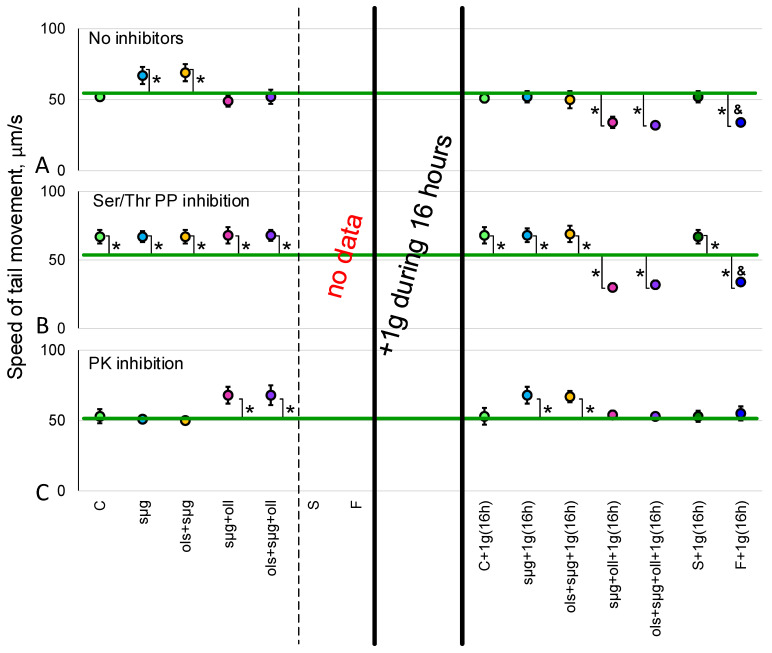
**The speed of movement of the end of the tail of the *Drosophila melanogaster* sperm.** (**A**)—without inhibitors. (**B**)—20 nM calyculin A (CA) was used as an inhibitor of Ser/Thr phosphatases. (**C**)—0.5 mM 6-DMAP was used as an inhibitor of the broad-spectrum protein kinases. *—*p* < 0.05 in comparison with the control group C; ^&^—*p* < 0.05 in comparison with the synchronous group S+1g(16h). Colors of points correlate with group colors of the experimental design schema (Figure 1). Speed values were not significantly different between control groups C, C+1g(16h), and S+1g(16h)—this level is marked with a green line. There were no data for the sperm tail speed in groups S and F due technical impossibility at the landing site.

In the 16 h after landing, in the F+1g(16h) group, the speed of movement of the end of the sperm tail (Figure 2A) was significantly reduced by 35% (*p* < 0.05) compared to the S+1g(16h) synchronous control group. The addition of Ser/Thr phosphatase inhibitor, calyculin A, led to an 29% (*p* < 0.05) increase in the speed of spermatozoa movement in the synchronous control group S+1g(16h); however, in the flight group F+1g(16h) the speed remained reduced (Figure 2B). The addition of a broad-spectrum protein kinase inhibitor did not lead to changes in movement speed in the S+1g(16h) group, but restored the motility speed of the F+1g(16h) group to the level of the control group (Figure 2C).

In the simulation experiment, the speed of movement of spermatozoa in the group of simulated microgravity sµg exceeded that in group C by 29% (*p* < 0.05). Similarly, the speed of spermatozoa movement was 33% (*p* < 0.05) higher in the ols+sµg group (simulated microgravity with overloads at launch) than in the control group. In the simulated microgravity group with landing overloads (sµg+oll), as well as in the microgravity group with take-off and landing overloads (ols+sµg+oll), the speed of spermatozoa did not differ from the control level (Figure 2A). Incubation with 20 nM calyculin A led to a 29% (*p* < 0.05) increase in movement speed in control group C (there were no differences from this level of control) as well as between groups of simulated microgravity (without and with launch overloads)—the speed was approximately 30% higher in all groups (Figure 2B). The addition of a broad-spectrum protein kinase inhibitor, 0.5 mM 6-DMAP, did not change the movement speed in group C; however, at the same time, in the sµg and ols+sµg groups, it neutralized the effect of increasing speed under simulated weightlessness. In contrast, speeds in groups with landing overloads sµg+oll and ols+sµg+oll increased and exceeded the level of group C in both cases by 28% (*p* < 0.05) (Figure 2C).

In the sµg+1g(16h) and ols+sµg+1g(16h) groups in simulated microgravity and with the addition of starting overloads, after 16 h of exposure to 1g conditions movement speed decreased (compared to the sµg and ols+sµg groups) and did not differ from the control. In groups with overloads on landing and a full flight sequence, sµg+oll+1g(16h) and ols+sµg+oll+1g(16h), movement speeds also dropped below the control level by 33% (*p* < 0.05) and 37% (*p* < 0.05), respectively (Figure 2A). The addition of calyculin A led to a 30% (*p* < 0.05) increase in speed in groups C+1g(16h), sµg+1g(16h) and ols+sµg+1g(16h). In groups with overloads on landing, sµg+oll +1g(16h) and ols+sµg+oll+1g(16h), as in the F+1g(16h) flight group, speeds did not increase and remained approximately 35% below the control level (*p* < 0.05) (Figure 2B). Incubation with a protein kinase inhibitor did not change the movement speed in the C+1g(16h) group, increased it by 28% (*p* < 0.05) in the sµg+1g(16h) and ols+sµg+1g(16h) groups, and restored the reduced movement speed spermatozoa to the control level in the sµg+oll+1g(16h) and ols+sµg+oll+1g(16h) groups, as well as the F+1g(16h) group (Figure 2C).

Meanwhile, the frequency of spermatozoa beats did not differ between study groups (Table 1).

### 2.3. Cellular Respiration and the Content of Proteins Involved in It

**Figure 3:**
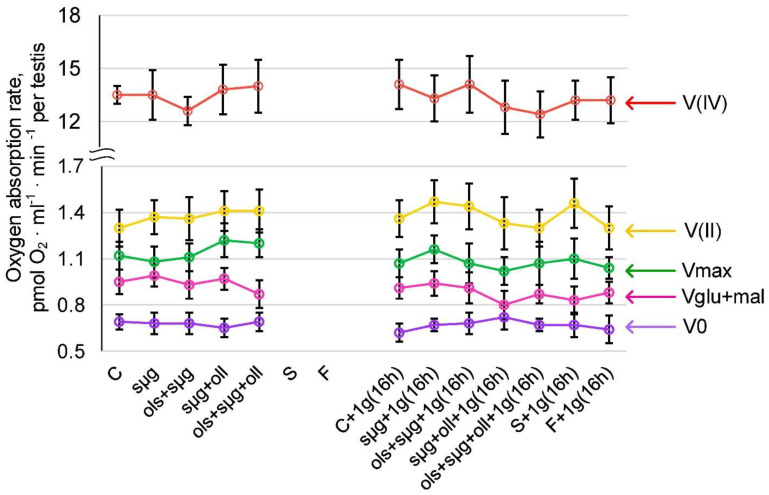
**Oxygen absorption rate by the testes of *Drosophila melanogaster*.** V0—oxygen absorption rate of permeabilized cells; Vglu+mal—oxygen absorption rate after adding 10 mM glutamate + 5 mM malate; Vmax—maximum rate after adding 2 mM ADP; V(II)—oxygen absorption rate after adding 0.5 μM rotenone (complex I inhibitor) and the subsequent supplement 10 mM succinate (substrate of complex II); and V(IV)—oxygen absorption rate after adding 5 μM antimycin (complex III inhibitor) and the subsequent supplement 0.5 mM TMPD + 2 mM ascorbate (artificial substrates of complex IV).

Likewise, in vivo studies of cellular respiration at the landing site were not possible. In the 16 h after landing, none of the parameters of cellular respiration (the rate of oxygen uptake by permeabilized cells V0, with the addition of the substrates of the first complex of the respiratory chain Vglu+mal; and the maximum respiration rate Vmax, as well as on the substrates of the second V(II) and fourth V(IV) respiratory chain complexes) in the F+1g(16h) group differed significantly from the S+1g(16h) group (Figure 3). In the simulation experiment, there were also no differences either immediately after the end of the experiment or after 16 h of readaptation to 1 g.

**Figure 4:**
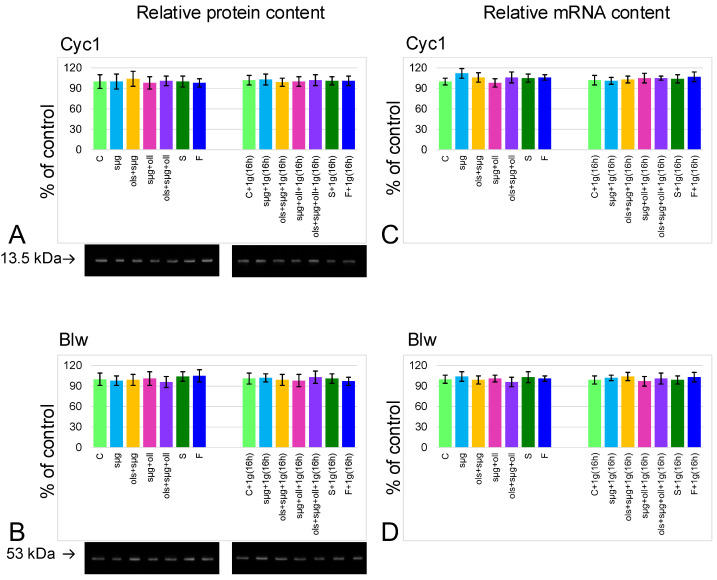
**Relative content of proteins, participation in cellular respiration, and their mRNA.** (**A**)—cytochrome *c* (Cyc1) protein with typical western-blot (MW 13.5 kDa). (**B**)—ATP synthase, H+ transporting, mitochondrial F1 complex, alpha subunit 1 (Blw, bellwether) with typical western-blot (MW 53 kDa). (**C**)—Cyc1 mRNA. (**D**)—Blw mRNA.

The relative content of both cytochrome *c* (Figure 4A) and the catalytic subunit of ATPsyntase (Figure 4B) did not change in all study groups relative to the control level; neither did the mRNA of the corresponding genes (Figure 4C,D).

### 2.4. Relative Content of the Main Cytoskeletal Proteins and mRNA of the Corresponding Genes

**Figure 5:**
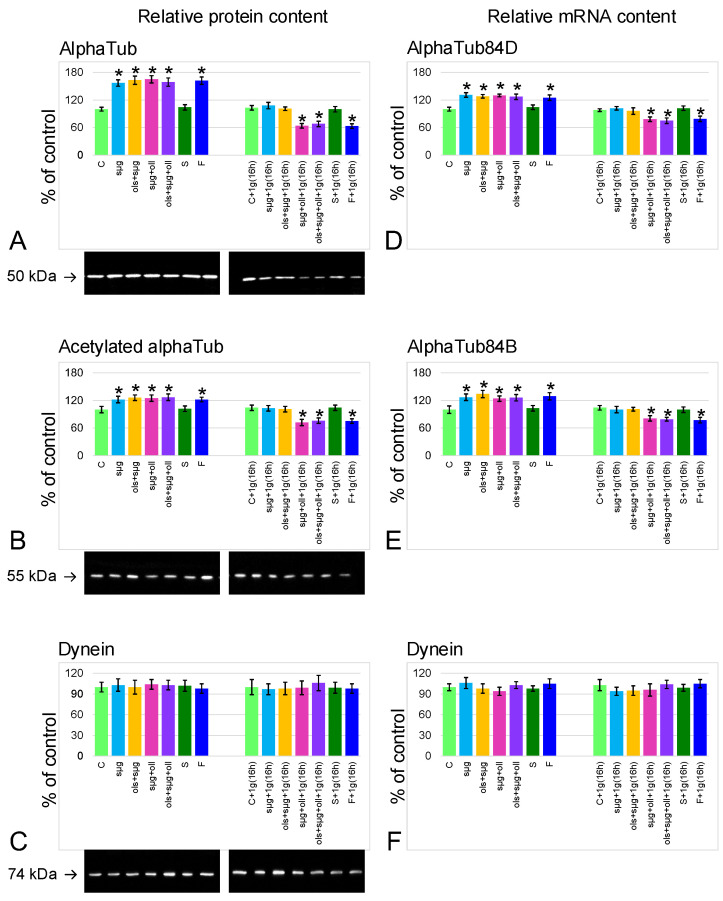
**Relative content of the cytoskeletal proteins, participation in the sperm motility, and theri mRNA.** (**A**)—alpha-tubulin (alphaTub) protein with typical western-blot (MW 50 kDa). (**B**)—acetylated alpha-tubulin protein with typical western-blot (MW 55 kDa). (**C**)—dynein protein content with typical western-blot (MW 74 kDa). (**D**)—alphaTub84D mRNA. (**E**)—alphaTub84B mRNA. (**F**)—dynein mRNA. *—*p* < 0.05 in comparison with the control group.

The relative alpha-tubulin content in groups that spent 12 days in simulated microgravity (sµg, ols+sµg, sµg+oll, and ols+sµg+oll) or spaceflight (F) was approximately 60% higher than in the control (*p* < 0.05) (Figure 5A). After exposure to the Earth’s gravity for 16 h, the relative content of alpha-tubulin decreased to the control level in groups without landing overloads (sµg+1g(16h) and ols+sµg+1g(16h); in groups with landing overloads (sµg+oll+1g(16h), ols+sµg+oll+1g(16h) and F+1g(16h)), it fell below the control level by 36% (*p* < 0.05), 32% (*p* < 0.05), and 37% (*p* < 0.05), respectively. The same dynamics were demonstrated by changes in the mRNA content of genes encoding alpha-tubulin isoforms 84D and 84B (Figure 5D,E).

The content of acetylated isoform changed in a similar way to alpha-tubulin (Figure 5B), but although the changes were less pronounced, they were significant: in the sµg, ols+sµg, sµg+oll, ols+sµg+oll, and F groups, it increased by 22% (*p* < 0.05), 26% (*p* < 0.05), 25% (*p* < 0.05), 27% (*p* < 0.05), and 22% (*p* < 0.05), respectively. In the sµg+1g(16h), ols+sµg+1g(16h), and ols+sµg+1g(16h) groups, the relative content of acetylated alpha-tubulin decreased to the control level; in the sµg+oll+1g(16h), ols+sµg+oll+1g(16h), and F+1g(16h) it fell below the control by 28% (*p* < 0.05), 24% (*p* < 0.05), and 25% (*p* < 0.05), respectively.

Against the background of changes in the content of tubulin, the relative content of the motor protein dynein (Figure 5C), as well as the mRNA of the corresponding gene (Figure 5F), does not change in all study groups.

## 3. Discussion

The realization of the reproductive function in the natural cycle in most animal species is impossible without pronounced spermatozoa motor activity. Therefore, maintaining and/or restoring the motility of male sperm cells is an urgent task in the exploration of deep space with the subsequent goal of preserving the species.

The most significant data can be obtained under the conditions of a real space flight, or immediately after it. However, organizational and technical difficulties often do not provide such opportunities. Therefore, in this series of studies, in parallel with the “Cytomehanarium” space experiment carried out under space flight conditions aboard the RS ISS, a simulation experiment was carried out to identify the contribution of various factors to changes in the motor activity of fly spermatozoa.

The results obtained indicate that after a 12-day space flight, which was naturally accompanied by overloads during the launch of the spacecraft into orbit and landing of the descent vehicle, as well as a 16 h readaptation to gravity, the speed of spermatozoa decreased by 35% (*p* < 0.05) relative to the control (Figure 2).

In contrast, the results of the simulation experiment show that remaining in simulated microgravity, even with the addition of starting overloads, leads to an increase in the movement speed of fly spermatozoa compared to the control, which we noted earlier in shorter experiments [22,23]; this was also shown for sea urchin spermatozoa [25,26]. In short duration experiments this effect is associated not with a change in phosphorylation [26], but, apparently, with an increase in dephosphorylation under weightless conditions [23]. However, in this case, when the full cycle of gametogenesis took place under real or simulated conditions, the situation does not seem as unambiguous.

At the same time, the addition of landing overloads neutralizes this effect; immediately after “landing”, the speed of spermatozoa movement is the same as in the control. It was previously shown that the effect of overloads leads to a decrease in the speed of spermatozoa movement [26,27,28]. This means that the absence of differences in speed in the sµg+oll and ols+sµg+oll groups from the control group cannot be considered as no effect. It can be assumed that the decrease in speed in these groups compared to the control level is a superposition of the effects of an increase in speed in weightlessness and a decrease in speed under the action of hypergravity. The subsequent 16 h exposure to conditions of 1g leads to a decrease in movement speed in all groups of the simulation experiment compared to the quasi “landing site”. In microgravity and microgravity groups with starting overloads, the speed decreases to the control level; in groups with landing overloads, the speed drops below the control level, and speed values do not significantly differ from data for the space flight group.

In other words, the observed decrease in the speed of movement of the spermatozoa of the fruit fly *Drosophila melanogaster* after a 12-day space flight and 16 h readaptation under the Earth’s gravity is due to the effect of hypergravity during the landing of the descent vehicle and a period of readaptation to 1 g after a long period in weightlessness.

The pathogenesis of a change in the speed of movement towards an increase in weightlessness and a subsequent decrease in the early period of readaptation may be associated with a change in the energy supply of the motor activity of spermatozoa; although earlier, in short experiments, we did not observe a decrease in cellular respiration [22]. However, for another ATP-dependent motor system, actin-myosin in the left ventricle of the heart, it was shown that under conditions of simulated microgravity of a similar duration (approximately 14 days), an increase in the intensity of cellular respiration occurs, which then decreases in the early period of readaptation [29]. Therefore, studies of the parameters of cellular respiration were undertaken.

The rate of oxygen uptake by permeabilized cells (V0 ) was determined by adding the substrates of the first respiratory chain complex Vglu+mal, the maximum respiratory rate Vmax, as well as the substrates of the second V(II) and fourth V(IV) complexes of the respiratory chain during inhibitory analysis. All these parameters did not significantly differ between study groups (Figure 3). There were also no significant differences in the content of respiratory chain proteins and the expression of the corresponding genes (Figure 4).

On the other hand, a change in the speed of movement may be associated with a combined change in the content of proteins that provide motility, and also with a change in their activity as a result of the regulation of phosphorylation/dephosphorylation. Therefore, the content of tubulin, its acetylated isoform as a marker of stable microtubules, and dynein as the main motor protein were determined (Figure 5). Phosphorylation of dynein is a factor regulating sperm motility; it can both activate and, conversely, reduce motor activity [30,31,32,33,34,35,36]. It was shown that under weightlessness conditions the content of microtubule components increases, while that of dynein does not change. After 10 min of hypergravity during the landing of the descent vehicle, we do not detect (using Western blotting) a change in the total content of these proteins compared to their total content under microgravity conditions. We can assume that the process of cytoskeleton change was already initiated. This assumption is based on the fact that in groups that were under conditions of 1 g for 16 h without overloads on landing (groups sµg+1g(16h) and ols+ sµg+1g(16h)), the content of microtubule proteins decreases to the control level, and in groups exposed to the action of landing overloads (groups sµg+oll+1g(16h), ols+sµg+oll+1g(16h), and F+1g(16h)) it decreases more dramatically, to a level significantly lower than the control level.

In other words, with any increase in external mechanical stress in the early period, a decrease in the content of cytoskeletal proteins occurs, both in the case of the action of 1 g for 16 h, and more significantly under the action of more stressful conditions—overloads during landing followed by the action of 1 g for 16 h. Change in the cytoskeleton structure as a result of changes in external mechanical conditions is a well-documented fact. Moreover, the transition from microgravity to the Earth’s gravity or from 1 g to hypergravity often leads to more pronounced disorders of cytoskeletal structures. In an experiment with mouse spermatozoa, we showed that under hypergravity conditions, motility decreases against the background of the destruction of the tubulin cytoskeleton, as well as after 1 h of exposure to 2 g conditions [27]. In addition, changes in tubulin content, especially in its acetylated form, may reflect changes in microtubule dynamics. Sperm tails are primary cilia, in which protein synthesis does not occur [37]. Synthesized proteins are transported from the cytoplasm to the axoneme via intraflagellar transport trains by motor proteins kinesin and dynein [38,39]. In addition to this pathway, passive diffusion occurs for approximately a third of alpha-tubulin molecules, which can be formed as a result of microtubule depolymerization [40]. At the same time, it is impossible to determine this using Western blotting under denaturing conditions, which is a limitation of this study.

**Figure 6:**
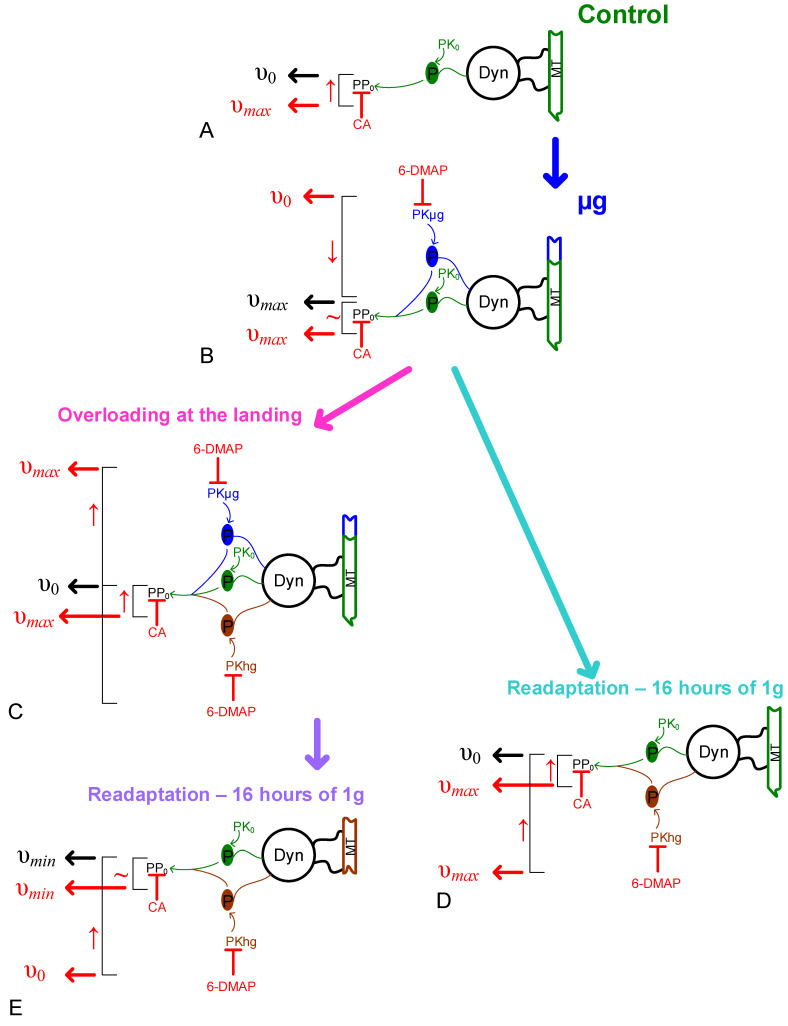
**A possible scheme for regulating the speed of movement of spermatozoa of the fruit fly *Drosophila melanogaster*, of which the full cycle of gametogenesis passed under the conditions of a space flight or a simulation experiment.** Green indicates events related to control, blue indicates microgravity action, and brown indicates hypergravity action. The action of inhibitors and their effects are indicated in red. Dyn—dynein; MT—microtubules; PK—protein kinases; PP—protein phosphatases; 6-DMAP—6-(dimethylamino)purine, broad-spectrum protein kinases inhibitor; and CA—calyculin A, Ser/Thr phosphatase inhibitor. See Figure 2 for speed values; υ_0_—movement speed in the control (approximately 50 µm/s); υ*_max_*—movement speed after the action of CA or microgravity (approximately 70 µm/s); and υ*_min_* —movement speed after overloading at the landing and subsequent readaptation to Earth gravity for 16 h (approximately 30 µm/s). (**A**)—phosphorylation of dynenin, Dyn is provided by the constitutive protein kinase activity PK_0_ (insensitive to the 6-DMAP inhibitor); and dephosphorylation—by the constitutive protein phosphatase activity, PP_0_, inhibition by which caliculin A (CA) leads to an increase in speed up to υ*_max_*. (**B**)—under microgravity conditions, the speed of movement increases to υ*_max_* due to the appearance of additional protein kinase activity, Pµg, which is inhibited by 6-DMAP, while speed decreases to the control level υ_0_. (**C**)—the short-term effect of landing overloads activates another protein kinase activity, PKhg, which leads to a decrease in speed to the control level υ_0_ (for possible examples of such protein kinases, see the Discussion text). Accordingly, inhibition of this activity by 6-DMAP, as well as a decrease in the protein phosphatase activity by CA, restores the rate to that of the level of microgravity conditions υ*_max_*. (**D**)—long-term exposure to 1 g under readaptation conditions leads to the cessation of additional protein kinase activity, Pµg, and the appearance of Phg activity. Similarly, the action of 6-DMAP and CA increase the speed to that of the level of microgravity. (**E**)—the combined effect of overloads during landing and the readaptation period (as in the post-flight group) leads to a decrease in speed to υ*_min_* against the background of activation of additional protein kinase activity, Phg, as well as destruction of the cytoskeleton. The use of a 6-DMAP inhibitor restores the speed of spermatozoa to the control level υ_0_.

The data regarding the content of the main proteins that form the spermatozoon axoneme allow interpretation of the results of tail movement measurements obtained using kinase and phosphatase inhibitors, revealing the role of phosphorylation/dephosphorylation regulation (Figure 6). Nevertheless, it should be noted that data for the length of spermatozoon tails can provide direct evidence of a change in the structure of the axoneme. However, direct measurements are difficult due to the long length of the tails and their confusion in the duct, which is a limitation of this study.

The application of the Tyr phosphatase inhibitor sodium orthovanadate leads to a complete cessation of motility in all study groups, which indicates that the dephosphorylation of Tyr residue is an absolutely necessary condition for the motor activity of *Drosophila melanogaster* spermatozoa.

The application of the Ser/Thr phosphatase inhibitor calyculin A leads to an increase in motility in the control to the same level as in simulated microgravity. The use of the protein kinase inhibitor 6-(dimethylamino)purine (6-DMAP) does not change the motility rate in the control, which indicates that phosphorylation under normal conditions is carried out by protein kinases that are not sensitive to 6-DMAP, despite its wide spectrum of action.

The application of calyculin A to samples after simulated microgravity does not change their speed—it remains at the same high level. However, the use of 6-DMAP leads to a leveling effect: the speed after simulated microgravity decreases to the control level. This may indicate that in spermatozoa, an additional protein kinase activity, sensitive to 6-DMAP, appears during the full cycle of formation and maturation that takes place under simulated weightlessness, which leads to an increase in the speed of movement. Protein kinases, the activity in spermatozoa that leads to an increase in the speed of movement, can be, for example, cAMP-dependent protein kinase A (PKA) [26,30], protein kinase C (PKC) (the activity of the latter being inhibited by the 6-DMAP analog), or staurosporine [41,42].

The action of “landing” overloads in the simulation experiment leads to a decrease in the speed of movement to the level of the control. Both calyculin A and 6-DMAP restore this rate to the level it was in microgravity. At the moment, we still do not notice changes in the content of proteins (as explained above). Therefore, it can be assumed that an increase in mechanical stress leads to an increase in protein phosphorylation, which inhibits locomotor activity (6-DMAP restores), and reduces phosphorylation, which activates locomotor activity (calyculin A restores). As the action of calyculin A is the same as that of the control, it is likely that the phosphatases are the same as well. Protein kinases, the action of which will lead to a decrease in the motor activity of spermatozoa, can be, for example, glycogen synthase kinase 3 (GSK3) [34] and phosphatidylinositol 3-kinase (PI3K) [33].

Similarly, when speed is reduced to the level of control as a result of the Earth’s gravity for 16 h, calyculin A and 6-DMAP contribute to its increase to the level of microgravity.

However, in the case of the combined action of hypergravity during the landing of the descent vehicle and the subsequent period of readaptation to the Earth’s gravity for 16 h, a decrease in the content of proteins that form the axoneme of the tail of the spermatozoon occurs. In this case, the action of calyculin A does not lead to an increase in speed; 6-DMAP increases speed, but to the level of control (not microgravity). In other words, the combined effect of overload and 16 h readaptation partially damages the structure that provides motility, and, against the background of an increase in inhibitory protein kinase and phosphatase activities, leads to a dramatic decrease in the rate.

Thus, in the spermatozoa of the fruit fly *Drosophila melanogaster*, the full cycle of gametogenesis which took place under microgravity conditions, additional activity of protein kinases appears, which activates the positive (for motility) phosphorylation of spermatozoon proteins. The action of hypergravity leads to an increase in the activity of protein kinases which activates the negative (for motility) phosphorylation of spermatozoon proteins, as well as to an increase in the activity of phosphatases. In the case of partial destruction of the microtubule cytoskeleton, the contribution of inhibitory protein kinase activity is decisive: the use of a 6-DMAP inhibitor facilitates an increase in the speed of movement.

## 4. Materials and Methods

### 4.1. Determination of the Parameters of the Sperm Motility

The speed of sperm movement was measured in accordance with the method described in detail by us earlier, including the use of phosphatase and kinase inhibitors [22,23].

Briefly, the testes of the flies of each study group were isolated and dissected in saline to extract the spermatozoa. Next, the inhibitor was added and incubated for 10 min. Sodium orthovanadate Na_3_VO_4_ (CAS 13721-39-6, Calbiochem, San Diego, CA, USA), at a working concentration of 200 μM, was used to inhibit tyrosine phosphatases [43,44]; for inhibition of serine/threonine phosphatases—calyculin A (# 19-139, Millipore, Merck, St. Luis, MO, USA) at a working concentration of 20 nM [45]; and for protein kinases—6-(dimethylamino) purine (6-DMAP) (ab145307, Abcam, Cambridge, UK) at a working concentration of 0.5 mM [46]. After 10 min incubation at room temperature, a drop of the obtained suspension with a volume of 5 μL was transferred into a Makler chamber (Sefi Medical Instruments Ltd., Haifa, Israel) and placed under a phase-contrast microscope objective (Eclipse E200 MV, Nikon, Tokyo, Japan) with a general 200× magnification. Videos of sperm movements were recorded at 60 frames per second with a resolution of 2 megapixels (Basler puA1600-60uc color camera with e2V EV76C570 CMOS sensor, Basler AG, Ahrensburg, Germany). Video images were analyzed using the Fiji software package with the Manual Tracking plugin (open access https://imagej.net/software/fiji/, accessed 6 April 2022). As before, the frequency of movement (in Hz) was determined as the number of crossings of a line perpendicular to the main axis of the tail, and the speed of movement (in μm/s) as the distance traveled by the end of the tail per second.

### 4.2. Determination of Cellular Respiration Parameters

Similarly, to determine the parameters of cellular respiration in tissue by polarography using inhibitory analysis [47] in the testes of the fruit fly after a space flight, the same technique was used as before [22].

For one biological replica, the testes of twenty flies from each study group were used. Results from at least three biological replicas were obtained for each study group. The testes were removed in saline, 10 μg/mL saponin was added, then incubated for 15 min at +22 °C and transferred to an Oxygraph+ polarographic cuvette (Hansatech Instruments Ltd., Norfolk, UK). The rate of oxygen uptake (in pmolO_2_/mL/min/testes) was measured using sequential addition of substrates and inhibitors: the rate of oxygen uptake by permeabilized cells, V_0_, 10 mM glutamate, and 5 mM malate (substrates of the first complex) were added, followed by Vglu+mal, then 2 mM ADP—Vmax, 0.5 μM rotenone (complex I inhibitor), 10 mM succinate (substrate of complex II)—V(II), then 5 μM antimycin A (complex III inhibitor), and 0.5 mM TMPD + 2 mM ascorbate (artificial substrates of complex IV)—V(IV). After the substrate-inhibitory analysis, each sample was tested for the intactness of the outer mitochondrial membrane by adding 10 μM cytochrome *c*; if the membrane was intact, then the respiratory rate did not change or increased by a maximum of 15%. If the membrane was not intact in the analyzed sample, then the results of this sample were not taken into account, and the replica was repeated.

### 4.3. Determination of the Relative Content of Proteins

For one biological replica, the testes of twenty flies from each study group were used. For each study group, the results of at least three biological replicas were obtained. The testes were isolated, immediately frozen, and stored at −60 °C until the proteins were isolated.

For protein isolation, frozen samples were homogenized in Laemmli buffer with the addition of a mixture of phosphatase inhibitors (1 mM sodium orthovanadate and 50 mM sodium fluoride) and proteinases (Calbiochem, San Diego, CA, USA). The concentration of total protein in the obtained samples was measured and the same amount was applied to each well of a polyacrylamide gel, in which electrophoresis was performed under denaturing conditions (Bio-Rad Laboratories, Hercules, CA, USA), and subsequently transferred to a nitrocellulose membrane [48], the effectiveness of which was controlled by Ponceau staining. After transfer, membranes were blocked in 4% milk (Skim milk powder #70166-500G, Sigma-Aldrich, Germany) and stained with primary antibodies (Table 2) and their corresponding HRP-conjugated secondary antibodies (anti-rabbit #7074S, anti- mouse #7076S, all Cell Signaling Technology, Danvers, MA, USA). Next, membranes were treated with substrates (SuperSignal™ West Femto Maximum Sensitivity Substrate, Thermo Scientific, Waltham, MA, USA), detected using ChemiDoc XRS+ imaging system (Bio-Rad Laboratories, Hercules, CA, USA) and processed using Image Lab Software (Bio-Rad Laboratories, Hercules, CA, USA).

### 4.4. Determination of Relative mRNA Content

For one biological replica, the testes of twenty flies from each study group were used. For each study group, the results of at least three biological replicas were obtained. The testicles were isolated, immediately frozen, and stored at −60 °C until mRNA isolation.

Total mRNA was isolated using the RNeasy Micro Kit (#74004, Qiagen, Hilden, Germany) according to the manufacturer’s instructions. Next, reverse transcription was performed with d(T)_15_ as a primer and 500 ng of RNA to obtain cDNA. Determination of the relative mRNA content of the studied genes was carried out using real-time PCR with specific primers (Table 3) and the results were obtained using the 2(-DeltaDeltaC (T)) method [49].

### 4.5. Statistical Analysis

The results were analyzed using two-way ANOVA using Student’s posterior t-test with Bonferroni’s correction for multiple comparisons. A significance level of *p* < 0.05 was used to assess the significance of changes. Data are presented as M ± SE (M is the mean and SE is the standard error of the mean).

## 5. Conclusions

Summarizing the results, we can conclude that in the early period of readaptation after space flight, the movement speed of the spermatozoa of the fruit fly Drosophila melanogaster decreases as a result of the action of overloads and subsequent exposure to 1 g after a long exposure to weightlessness. At the same time, cellular respiration, the content of respiratory chain proteins, and the expression of their genes all remain at the control level. The observed decrease in speed may be due to the activation of additional protein kinase activity against the background of a decrease in the relative content of tubulin. The use of a broad-spectrum protein kinase inhibitor 6-(dimethylamino)purine makes it possible to restore the parameters of the motor activity of the spermatozoa of the fruit fly Drosophila melanogaster to the control level after 16 h of exposure to Earth’s gravity after a 12-day space flight. The fundamental mechanisms of regulation of the motor activity of spermatozoa in space flight require further study; however, the data obtained may be useful in developing approaches to maintaining reproductive potential during deep space exploration.

## Figures and Tables

**Table 1:** Frequency (in Hz) of sperm tail movement.

	C	sµg	ols+sµg	sµg+oll	ols+sµg+oll	S	F
no inhibitors	1.54 ± 0.15	1.48 ± 0.10	1.51 ± 0.12	1.46 ± 0.13	1.50 ± 0.14	no data	no data
200 µM Na_3_VO_4_	0	0	0	0	0	no data	no data
20 nM CA	1.49 ± 0.16	1.49 ± 0.11	1.51 ± 0.17	1.49 ± 0.16	1.51 ± 0.11	no data	no data
0.5 mM 6-DMAP	1.56 ± 0.11	1.54 ± 0.09	1.51 ± 0.11	1.54 ± 0.13	1.59 ± 0.09	no data	no data
	**C** **+1g(16h)**	**sµg** **+1g(16h)**	**ols+sµg** **+1g(16h)**	**sµg+oll** **+1g(16h)**	**ols+sµg+oll** **+1g(16h)**	**S** **+1g(16h)**	**F** **+1g(16h)**
no inhibitors	1.48 ± 0.13	1.47 ± 0.13	1.50 ± 0.15	1.50 ± 0.11	1.48 ± 0.13	1.57 ± 0.11	1.54 ± 0.08
200 µM Na_3_VO_4_	0	0	0	0	0	0	0
20 nM CA	1.49 ± 0.15	1.47 ± 0.17	1.52 ± 0.16	1.54 ± 0.13	1.53 ± 0.16	1.48 ± 0.13	1.49 ± 0.13
0.5 mM 6-DMAP	1.56 ± 0.12	1.52 ± 0.17	1.59 ± 0.12	1.54 ± 0.11	1.58 ± 0.09	1.57 ± 0.10	1.58 ± 0.12

**Table 2:** Primary antibodies.

Protein	Manufacturer with Catalog Number, Dilution
cyt c-1 (cytochrome c-1, 13.5 kDa)	Abcam, Cambridge, UK, #ab13575, 1 µg/mL
blw (bellwether, ATP synthase mitochondrial F1 complex, alpha subunit 1, 53 kDa)	Abcam, Cambridge, UK, #ab14748, 1 µg/mL
aTub (alpha-tubulin, 50 kDa)	Abcam, Cambridge, UK, #ab52866, 1:10,000
acet-aTub (acetylated alpha-tubulin, 55 kDa)	Santa Cruz Biotechnology, Inc., Dallas, TX, USA, #sc-23950, 1:100–1:1000
dynein (axonemal, intermediate chain 1, 74 kDa)	Invitrogen by Thermo Fisher Scientific, Waltham, MA, USA, eBioscience^TM^ #14-9772-80, 5 µg/mL

**Table 3:** Primer sequences and product sizes.

Gene	Primer Sequence, Forward/Reverse (5′…3′)	Product Size, bp
*cyt c-1*	gcagcgacattgcgaagatt/actgctccagggcgtagata	181
*blw*	aataggagtagcggtgcgtg/aaccacggattgaaggcgat	201
*alphaTub84D*	aaggactacgaggaggtcgg/atgcgagtgggagcgtatga	124
*alphaTub84B*	cactggtacgttggtgaggg/cccatcgagcgttgaagtgg	166
*dynein*	agcaggtgaagggaaaaagg/accacgagattgcgaacgat	208
*gapdh*	atactcatcaaccctccccc/ggctgagttcctgctgtctt	142

## Data Availability

All data generated or analyzed during this study are included in this article.
